# Recognizing KBG syndrome in pediatric practice: a case series highlighting phenotypic variability and diagnostic clues

**DOI:** 10.3389/fped.2026.1850895

**Published:** 2026-07-27

**Authors:** Mai Labani, Zuhair Rahbeeni

**Affiliations:** Medical Genomics Department, King Faisal Specialist Hospital and Research Centre, Riyadh, Saudi Arabia

**Keywords:** *ANKRD11*, case series, craniofacial dysmorphism, genotype–phenotype correlation, intellectual disability, KBG syndrome, macrodontia, neurodevelopmental disorder

## Abstract

**Background:**

KBG syndrome (KBGS) is a rare autosomal dominant neurodevelopmental disorder caused by heterozygous pathogenic variants in ANKRD11. It is characterized by developmental delay, intellectual disability, behavioral difficulties, macrodontia, craniofacial dysmorphism, short stature, and skeletal anomalies.

**Objectives:**

To describe the clinical, radiological, and molecular findings of Saudi patients with molecularly confirmed KBGS and compare their presentation with reported features in the literature.

**Methods:**

We conducted a retrospective case series at King Faisal Specialist Hospital and Research Centre, Riyadh, between January 2002 and December 2024. Clinical, dysmorphological, developmental, behavioral, family history, neuroimaging, and molecular data were reviewed for three pediatric patients with confirmed *ANKRD11*-related KBGS.

**Results:**

All patients demonstrated core features of KBGS, including intellectual disability, speech and motor delay, learning difficulties, and behavioral manifestations such as autism spectrum features or aggression. Common dysmorphic findings included long philtrum and prominent ears in all patients, with a triangular face, macrodontia, and synophrys observed in two patients. Short stature and skeletal anomalies were also present. Molecular testing identified heterozygous pathogenic or likely pathogenic *ANKRD11* variants: NM_013275.6:c.4087C > T (p.Arg1363Ter), NM_013275.6:c.3382_3383del (p.Asp1128GlufsTer41), and NM_013275.6:c.1977C > G (p.Tyr659Ter). All variants were predicted to result in premature termination. Renal fusion and complex vertebral anomalies were observed, suggesting possible underrecognized systemic involvement, although cautious interpretation is required given the small sample size.

**Conclusion:**

This Saudi case series adds to the growing evidence of clinical heterogeneity in KBGS. Early recognition, molecular confirmation, multidisciplinary care, and longitudinal follow-up are essential to improve diagnosis, surveillance, and management.

## 1 Introduction

KBG syndrome (KBGS) is a rare autosomal dominant neurodevelopmental disorder caused by heterozygous pathogenic variants in the *ANKRD11* gene (OMIM 611192), located on chromosome 16q24, caused by haploinsufficiency of *ANKRD11*, a gene that plays a critical role in transcriptional regulation and chromatin remodeling (1, 2). Herrmann proposed that the name KBG represents the initials of the surnames of the first three unrelated families diagnosed with this disorder. The three letters correspond to the last names of the affected index families, commonly denoted in the literature as K, B, and G, and, as reported in 1975**,** the actual surnames behind the letters remain undisclosed in the public literature (3). KBGS is characterized by a recognizable constellation of clinical features, including intellectual disability, developmental delay, short stature, macrodontia, and distinctive craniofacial dysmorphism such as a round face and brachycephaly. Skeletal anomalies involving the spine, ribs, and limbs are also reported (4, 5).

In addition to structural abnormalities, a distinct neurobehavioral profile has been increasingly recognized, including features of autism spectrum disorder, attention deficits, and behavioral disturbances (6). Neuroimaging findings may include cerebellar vermis hypoplasia, corpus callosum abnormalities, brain calcifications, and optic nerve hypoplasia, reflecting the broad neurological involvement associated with *ANKRD11* dysfunction (7).

Phenotypic expression in KBG syndrome appears to evolve with age, with some features becoming more recognizable over time while others may diminish or normalize, potentially contributing to delayed diagnosis (8).

Since its initial description, the phenotypic and molecular spectrum of KBGS has expanded considerably, with growing recognition of both typical and atypical presentations (5, 9).

Due to its multisystem involvement, affected individuals often require multidisciplinary care and long-term surveillance. General management includes developmental and behavioral support, speech and occupational therapy, regular neurological follow-up for seizure assessment, hearing and ophthalmologic evaluations, dental care for characteristic craniofacial abnormalities, and endocrinology assessment for growth concerns (10–12). Long-term outcomes remain variable, with many patients demonstrating persistent cognitive and behavioral difficulties; however, most benefit from early supportive interventions and individualized educational programs (6, 13, 14).

In this study, we describe the clinical and molecular findings of three individuals with pathogenic variants in *ANKRD11*, associated with KBG syndrome. We highlight the phenotypic variability observed in this first Saudi cohort and contribute to the growing evidence on the broad clinical spectrum of KBG syndrome, including renal and vertebral manifestations that have been infrequently described in previous reports; however, renal fusion appears to be rarely described in the literature and may add to the expanding spectrum of genitourinary manifestations associated with the condition. Although limited by the small sample size, our findings suggest continued phenotypic heterogeneity in KBG syndrome and emphasize the importance of comprehensive clinical evaluation in affected individuals.

## 2 Objectives

- To characterize the clinical and molecular spectrum of KBG syndrome in the Saudi case series.
- To identify phenotypic variability and expand the recognized clinical spectrum through comparison with existing literature.

## 3 Materials and methods

This retrospective case series on the clinical and molecular characterization of KBG syndrome was conducted at King Faisal Specialist Hospital and Research Centre (KFSHRC), Riyadh, Saudi Arabia, between January 1, 2002, and December 31, 2024. Cases were identified through a referral-based approach from the Neurology and Medical Genetics clinics, in addition to review of the institutional molecular genetics database and electronic medical records.

Inclusion criteria included patients of any age or sex with a molecularly confirmed diagnosis of KBG syndrome. Exclusion criteria included patients with clinically suspected KBG syndrome without molecular confirmation.

Clinical data were retrospectively collected from electronic medical records and family interviews and included demographic characteristics, developmental milestones, neurobehavioral manifestations, dysmorphic features, skeletal abnormalities, imaging findings, and other systemic manifestations.

Molecular diagnosis was established using either whole exome sequencing (WES) or whole genome sequencing (WGS) with copy number variant (CNV) analysis. Genetic testing was performed in accredited molecular laboratories, including the Molecular Genetics Laboratory at KFSHRC, while one case was analyzed in an international reference laboratory prior to the establishment of local molecular diagnostic services at KFSHRC. Variants were interpreted according to standard clinical variant interpretation guidelines, and clinically significant variants identified in the index cases were validated using approved confirmatory methods.

Data analysis was conducted using a descriptive qualitative approach due to the small sample size and descriptive nature of the study. Clinical and molecular findings were summarized to characterize the phenotypic spectrum of KBG syndrome and compared with previously published literature to explore possible genotype–phenotype associations.

Written informed consent for genetic testing was obtained from the parents or legal guardians at the time of sample collection.

### 3.1 Patients' description

#### 3.1.1 Patient 1

**Figure 1:**
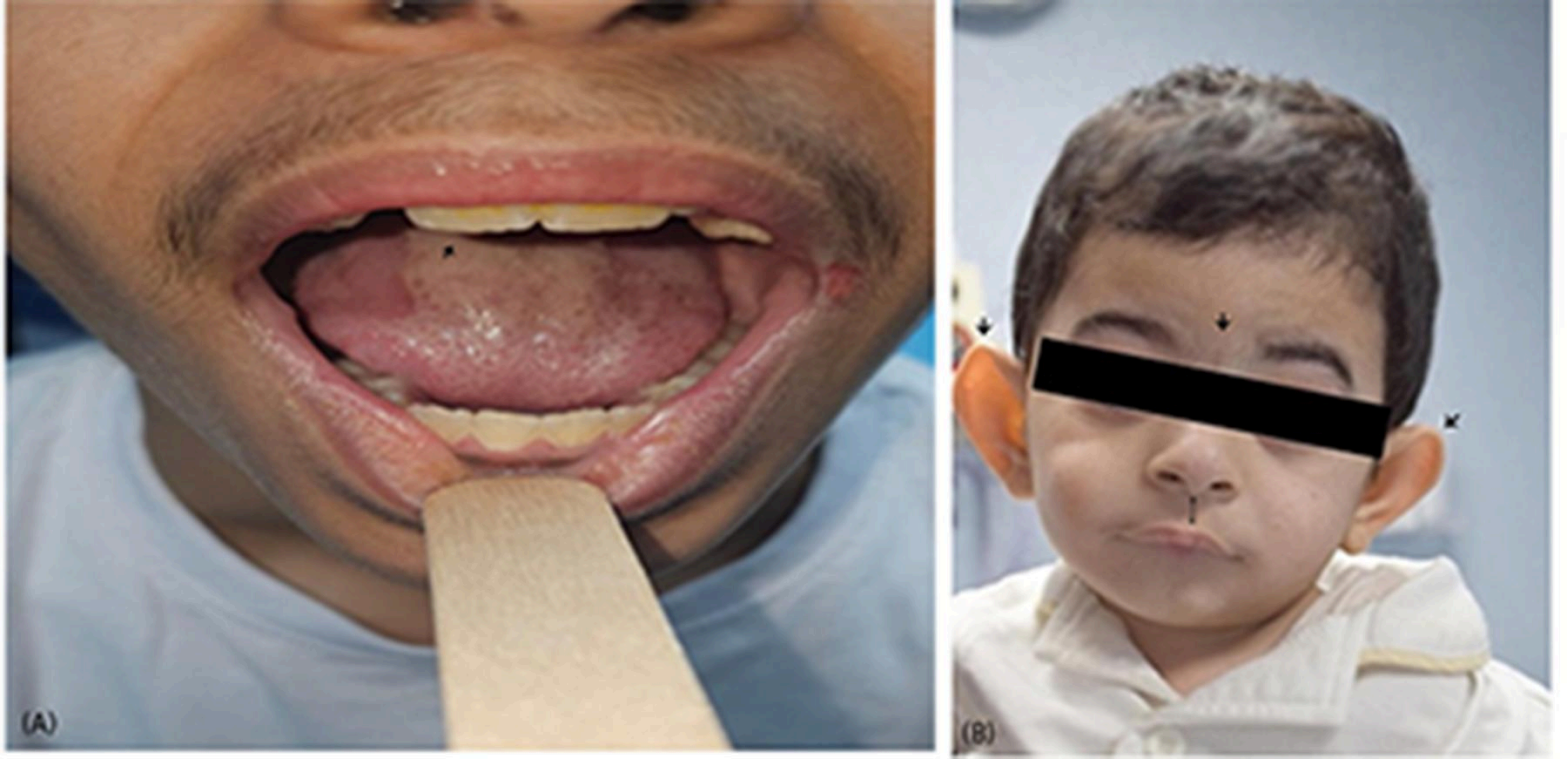
Clinical craniofacial and dental findings in patients with KBG syndrome. **(A)** Macrodontia with broad upper central incisors and prominent ridging of the permanent central incisors in Patient 1 (Black arrow). **(B)** Craniofacial features of KBG syndrome in Patient 2, including a smooth long philtrum, synophrys, and prominent ears (Black arrow).

A 14-year-old male, born full-term by cesarean section with no reported perinatal complications, was referred for global developmental delay, dysmorphic features, complex partial seizure disorder, and behavioral concerns. He had a significant developmental delay, sitting independently at 14 months and walking at 3 years. According to parental report, he began producing speech-like vocalizations at around 1 year of age; however, his verbal output remains unintelligible, and he engages in non-meaningful spoken language. He can feed himself but is not toilet-trained. Cognitively, he recognizes his family members but shows minimal interaction with others and displays features consistent with autism spectrum disorder, including impaired social engagement and aggressive behavior. On examination, his weight and height were below the 3rd percentile, while head circumference was at the 25th percentile. Dysmorphic features included a triangular face, hypertelorism, long philtrum, large prominent ears, synophrys, macrodontia with prominent ridging of the permanent central incisors, broad bushy eyebrows, anteverted nares, and hypoplastic alae nasi (Figure 1A). Family history revealed first-cousin consanguineous parents from an endogamous extended family structure. The patient has one healthy 16-year-old full sister and eight healthy half-siblings from a polygamous family setting. There is no known family history of a similar phenotype. Notably, the mother is affected by a mental health illness, and the child is currently being raised by his father and stepmother. Brain MRI showed nonspecific findings, including a relatively small brain volume compared to the cranial vault and prominent cerebrospinal fluid spaces for age, though white matter appeared adequately myelinated without focal abnormalities. Audiological assessment indicated mild to moderate conductive hearing loss in the right ear and slight to mild conductive hearing loss in the left ear. The electroencephalogram (EEG) was abnormal (grade III across awake, drowsy, and sleep states), with frequent spikes and sharp waves in the right temporooccipital region, generalized spike-and-polyspike-wave complexes, and intermittent slow activity in the same region, which is associated with complex partial seizure disorder.

#### 3.1.2 Patient 2

A 3-year-old Saudi boy, born at term via normal vaginal delivery to healthy consanguineous parents, required a one-week NICU admission for transient tachypnea of the newborn. He presented with global developmental delay and autistic-like features, including poor eye contact, limited social interaction, and stereotypic movements. He sat unassisted at 16 months, crawled at 22 months, and walked independently at 3 years, consistent with significant gross motor delay. He produced babbling sounds at around 1 year of age, with no history of language regression. His language development subsequently remained delayed, with persistently unintelligible verbal output. He also had learning difficulties. There was no history of seizures, and he was not receiving regular medications. Family history revealed first-cousin consanguineous parents from a monogamous endogamous marriage within an extended family structure. A maternal cousin was reported to have a mental health disorder; however, no additional similarly affected family members were identified. The physical examination showed short stature, hypotonia, diminished or sluggish reflexes, and dysmorphic features, including a smooth, long philtrum, macrodontia, synophrys, prominent ears, and clinodactyly; the remaining systemic examination was unremarkable (Figure 1B). Brain MRI and metabolic workup, including acylcarnitine, amino acid, and urine organic acid profiles, were normal.

#### 3.1.3 Patient 3

A 24-year-old female with learning difficulties and dysmorphic features was referred from the genetics clinic. Her early development was reportedly normal, but she later exhibited poor academic performance and failure to thrive. Dysmorphic features included a triangular face, long philtrum, micrognathia with a thin upper lip, large cupped ears, bilateral finger pads, crowded teeth, and synophrys. She had scoliosis requiring spinal fixation and asymptomatic bilateral renal fusion. On examination, weight and height were both below the 3rd percentile. Skin findings included hyperpigmentation of the proximal nail folds and nail thinning. Systemic and neurological examinations were otherwise unremarkable. Family history was significant for first-cousin consanguineous parents from a monogamous endogamous marriage within an extended family structure. She has eight siblings who are alive and well. One sibling died at 7 months of age due to cardiomyopathy. No other relatives with a similar phenotype were reported. The investigation included a US of the abdomen, which revealed partly fused bilateral duplex kidneys, suggestive of supernumerary kidneys versus two large kidneys with fullness of the renal pelvises; however, no hydronephrosis, focal lesions, or stones were identified.

CT Brain and EEG were unremarkable.

The family was not interested in proceeding with further segregation analysis.

Patients' demographics and family history are summarized in (Table 1).

**Table 1:** Demographic characteristics and family history.

	Current cases		
	Case 1	Case 2	Case 3
Demographic data			
Region	Eastern	Western	Central
Age of diagnosis	12 year	2 year	16 year
Current Age	14 year	3 year	24 year
Gender	Male	Male	Female
Growth parameters			
Current weight	3rd%ile	3rd%ile	3rd%ile
Current height	3rd%ile	3rd %ile	3rd%ile
Head circumference, if any	25th%ile	25th%ile	10th%ile
Family history			
Are the parents consanguineous?	Yes	Yes	Yes
Degree of relationship	first cousins	first cousins	first cousins
Is there a family history of similar symptoms?	No	No	No

## 4 Results

### 4.1 Clinical findings

In our analysis of the clinical findings in KBG syndrome, we began with a retrospective review of our case series, then integrated our data with previously published cases to better estimate the frequency of clinical features among affected individuals.

The age distribution in our study may also inform clinical observations, the developmental course, and the possible long-term prognosis of KBG syndrome, although definitive conclusions regarding long-term outcomes cannot be drawn from a limited number of adult patients.

Developmental and behavioral characteristics were consistently observed across all three patients in this KBG syndrome case series from Saudi Arabia. All patients exhibited intellectual disability, speech and motor delays, and learning difficulties. The average age at first reported speech-like vocalizations was approximately 1 year, while independent walking was delayed, occurring between 2 and 3 years of age. Social interaction challenges were universally present (3/3, 100%), with a formal diagnosis of autism spectrum disorder (ASD) documented in two cases (2/3, 66.6%), while aggressive behavior was reported in one case (1/3, 33.3%). These findings highlight the high burden of neurodevelopmental and behavioral impairments in individuals with KBG syndrome, as detailed in (Table 2).

**Table 2:** Neurodevelopment and behavioral characteristics.

	Current cases			Total cases (including current)	
	Case 1	Case 2	Case 3	Case/out of	%
Behavioral characteristics					
Autism spectrum disorder features	Yes	Yes	No	2/3	66.66
Aggressive behavior	Yes	No	No	1/3	33.3
Social interaction challenges	Yes	Yes	Yes	3/3	100
Developmental assessment					
Age at first words	1 year	1 year	1 year		
Age at walking	3 year	3 year	2 year		
Intellectual disability	Yes	Yes	Yes	3/3	100
Speech delay	Yes	Yes	Yes	3/3	100
Motor delay	Yes	Yes	Yes	3/3	100
Learning difficulties	Yes	Yes	No	3/3	100

**Figure 2:**
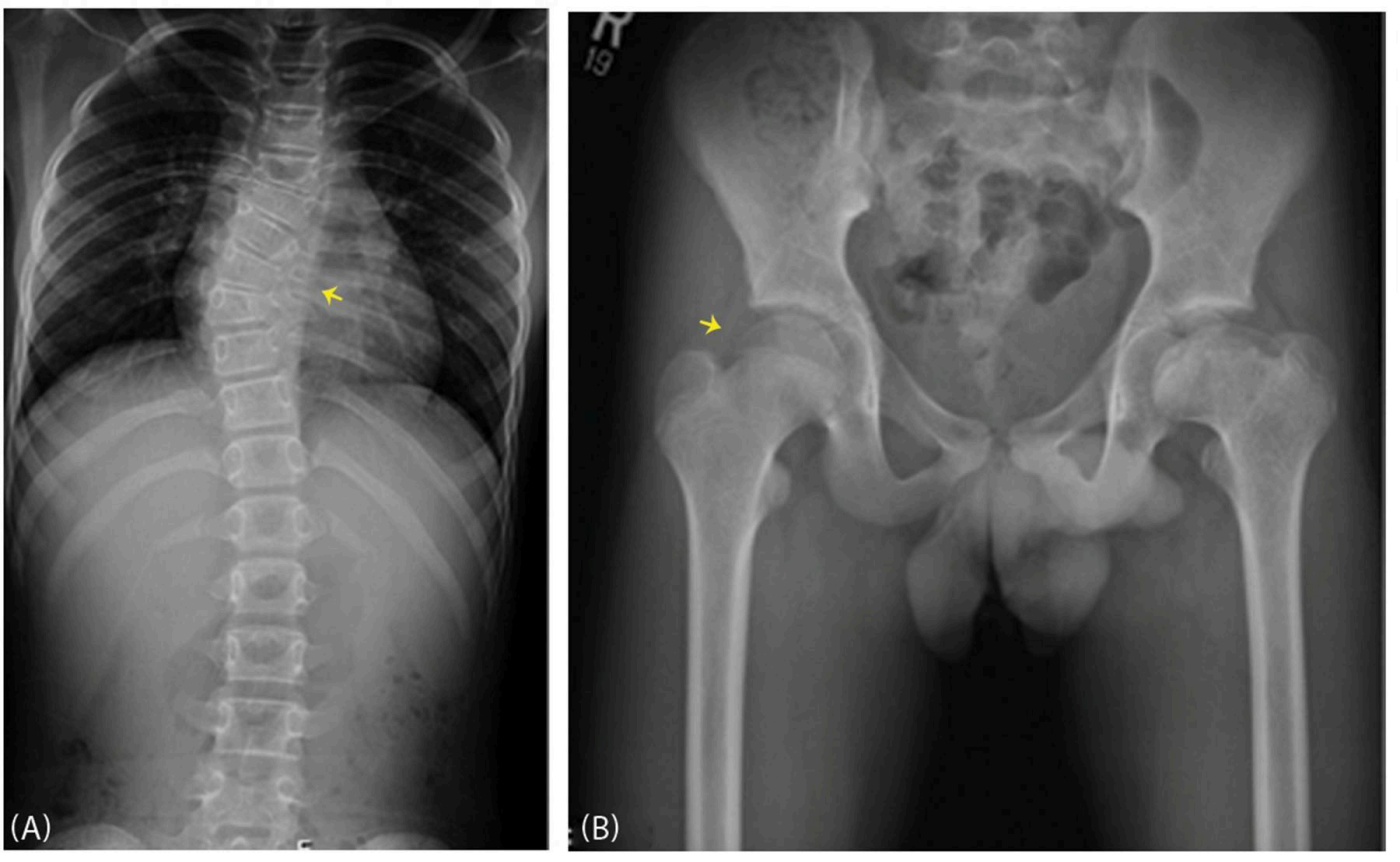
Radiographic skeletal findings in Patient 3 with KBG syndrome. **(A)** Anteroposterior thoracolumbar spine x-ray demonstrating right thoracic scoliosis with a suspected butterfly vertebra in the mid-thoracic region (yellow arrow). **(B)** Pelvic x-ray showing bilateral femoral head widening, more pronounced on the right side, with associated areas of sclerosis (yellow arrow).

In our national case series of KBG syndrome, all three patients demonstrated hallmark clinical features consistent with the diagnostic spectrum. The most consistent major features included a triangular face (66.6%), prominent ears (100%), a long philtrum (100%), and a delayed bone age (100%). Other common findings were macrodontia (66.6%), synophrys (66.6%), arched eyebrows (66.6%), a thin or long upper lip (66.6%) and hearing loss (33.3%), were also noted. Skeletal anomalies were present in varying forms among cases, including short or webbed neck (66.6%), clinodactyly (33.3%), and spinal abnormalities (33.3%), as noted. Focal dextroscoliosis centered at the mid-to-lower dorsal spine from T4 through T10 level with a Cobb angle of 25 degrees. There were multilevel partial vertebral fusion anomalies, a butterfly vertebra suspected between D4 and D5, and bilateral femoral head widening (33.3%) (Figures 2A,B).

According to current diagnostic criteria, KBG syndrome is typically diagnosed by identifying a heterozygous pathogenic variant in the *ANKRD11* gene. Alternatively, the 2023 clinical criteria proposed by Elena Martinez-Cayuelas require either a history of neurodevelopmental delay (motor and/or language), intellectual disability, attention-deficit/hyperactivity disorder (ADHD), and/or autism spectrum disorder (ASD), in combination with at least three phenotypic features, or fewer than three features accompanied by at least one of the main comorbidities (15), as summarized in (Table 3).

**Table 3:** Phenotypic and clinical features of KBG syndrome.

Clinical characteristics	% of KBG Patients (Counted individuals)			Total cases (Including current)		Literature frequency%
Phenotype	Case 1	Case 2	Case 3	Case/out of	%	
Craniofacial shape finding						
Triangular face	Yes	No	Yes	32/3	66.6	≈ 60–80%^12^
Any eyebrow finding						
Broad/wide eyebrows	Yes	No	No	1/3	33.3	≈30–50%^2^
Arched eyebrows	Yes	No	Yes	2/3	66.6	≈50–70^2^
Synophrys	Yes	Yes	No	2/3	66.6	≈50–70^2^
Any eye finding						
Hypertelorism and/or telecanthus	Yes	No	No	1/3	33.3	≈30–40^2^
Epicanthic folds Slanted palpebral fissures	No	Yes	Yes	2/3	66.6	≈40–60^2^
Any ear finding						
Protruding/prominent	Yes	Yes	Yes	3/3	100	≈70–80^2^
Any nose finding						30^2^
Upturned nose/anteverted	Yes	No	No	1/3	33.3	≈20–30^2^
Hypoplastic ala nasi	Yes	No	No	1/3	33.3	≈70–90^2^
Any mouth finding						
Thin or long upper lip	No	Yes	Yes	2/3	66.6	≈60^2^
High arched palate	No	Yes	No	1/3	33.3	≈30–40^2^
Long philtrum Flat/hypoplastic philtrum	Yes	Yes	Yes	3/3	100	≈70–90^2^
Any teeth finding						
Macrodontia of central incisors	Yes	Yes	No	2/3	66.6	≈85–90^12^
Malposition of teeth	Yes	No	No	1/3	33.3	≈30^2^
Skeletal anomalies						
5th finger clinodactyly	No	Yes	No	1/3	33.3	≈40^2^
Abnormal spine curvature	No	No	Yes	1/3	33.3	≈30–40^2^
Short femoral necks/hip dysplasia	No	No	Yes	1/3	33.3	Rare^2^
Delayed bone age	Yes	Yes	Yes	3/3	100	≈75^12^
Short and/or webbed neck	No	Yes	Yes	2/3	66.6	≈50^2^
Main comorbidities						
Hearing loss	Yes	No	No	1/3	33.3	≈20–30^2^
Seizure/ Epilepsy	Yes	No	No	1/3	33.3	≈26^18^
Bilateral renal fusion	No	No	Yes	1/3	33.3	Rare; specific frequency not well established^12,17^

### 4.2 Molecular findings

#### 4.2.1 Patient 1

Whole-exome sequencing (WES) through the Center for Genomic Medicine at King Faisal Specialist Hospital and Research Center in Riyadh, Saudi Arabia, showed a heterozygous nonsense variant in *ANKRD11* gene: NM_013275.6:c.4087C > T (p.Arg1363Ter), corresponding to chr16:89282455C > T (GRCh38). This variant introduces a premature termination codon and was classified as pathogenic.

#### 4.2.2 Patient 2

Whole genome sequence (WGS) through the Center for Genomic Medicine at King Faisal Specialist Hospital and Research Center in Riyadh, Saudi Arabia, showed a heterozygous likely pathogenic variant in *ANKRD11* gene: NM_013275.6:c.3382_3383del (p.Asp1128GlufsTer41). This variant was classified as likely pathogenic.

#### 4.2.3 Patient 3

Whole-exome sequencing and CNV analysis identified a heterozygous pathogenic variant in the *ANKRD11* gene: NM_013275.6:c.1977C > G (p.Tyr659Ter). This variant introduces a premature termination codon and was classified as pathogenic.

Molecular variants identified in the patients are summarized in (Table 4), which highlights the spectrum of *ANKRD11*-related KBG syndrome.

**Table 4:** *ANKRD11* mutations and micro arrangements found in the present series of KBG patients.

Test	Sex	*ANKRD11* variant	Exon	Zygosity	Variant Type	Classification
WES	Male	NM_013275.6:c.4087C > T (p.Arg1363Ter)	9	Heterozygous	Nonsense variant	Pathogenic
WGS	Male	NM_013275.6:c.3382_3383del (p.Asp1128GlufsTer41)	9	Heterozygous	Frameshift variant	Likely Pathogenic
WES and CNV	Female	NM_013275.6:c.1977C > G (p.Tyr659Ter)	9	Heterozygous	Nonsense variant	Pathogenic

WES, whole-exon sequence; WGS, whole-genome sequence; CNV, copy number variations.

**Figure 3:**
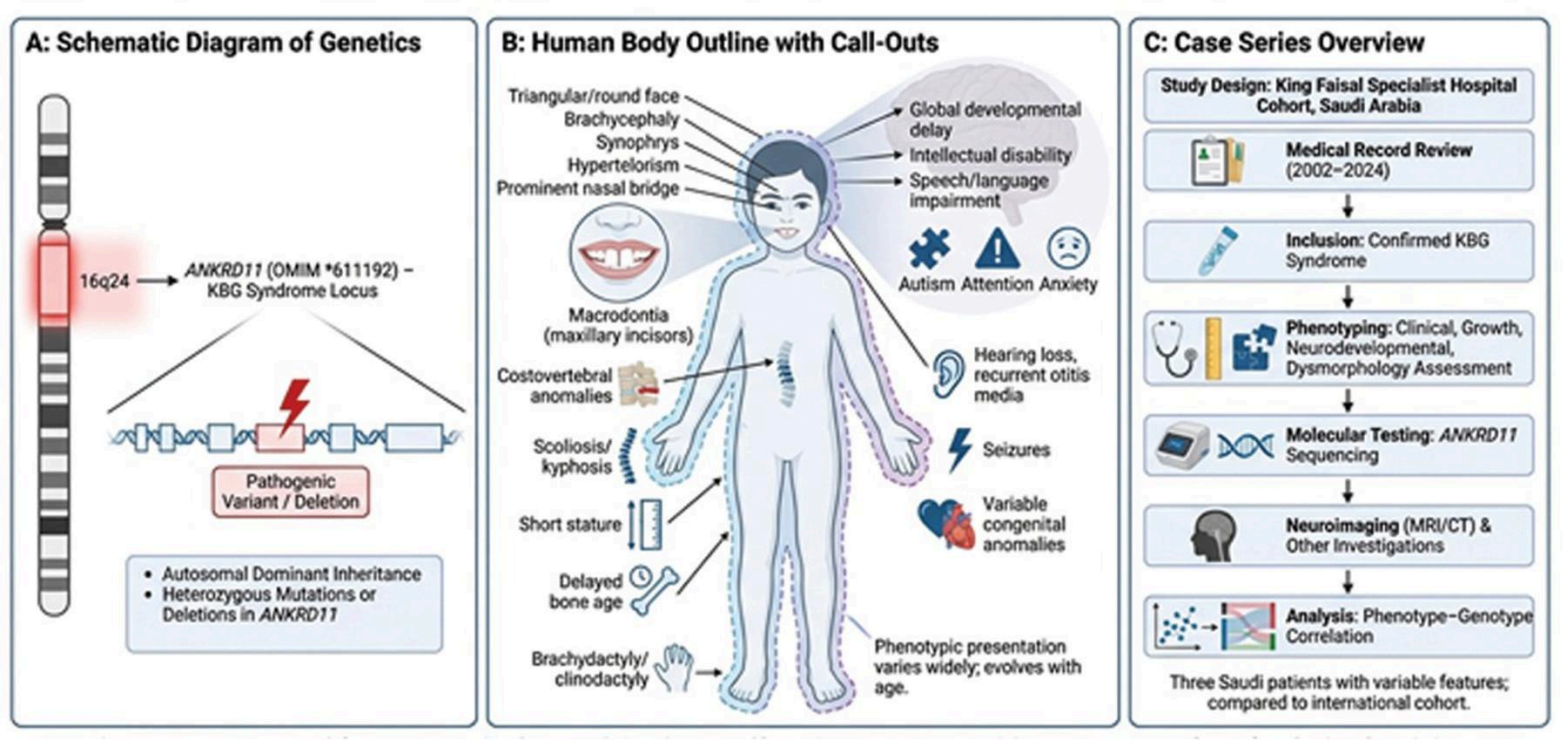
Image summarizing genetic and clinical overview of KBG syndrome: From *ANKRD11* variants to phenotypic Spectrum. **(A)** Schematic representation of chromosome 16 showing the 16q24 KBG syndrome locus and the *ANKRD11* gene, with pathogenic variants or deletions causing autosomal dominant KBG syndrome. **(B)** Summary of the characteristic clinical manifestations, including craniofacial features, macrodontia, global developmental delay, intellectual disability, speech impairment, behavioral abnormalities, hearing loss, seizures, short stature, delayed bone age, and skeletal and congenital anomalies. **(C)** Overview of the retrospective case‐series workflow, including medical record review, patient inclusion, clinical phenotyping, *ANKRD11* molecular testing, neuroimaging, and phenotype–genotype analysis of three Saudi patients.

An overview of *ANKRD11*-related KBG syndrome is illustrated in (Figure 3), demonstrating the genetic basis of the disorder, caused by pathogenic variants or deletions in *ANKRD11* on chromosome 16q24, along with the characteristic clinical manifestations, including craniofacial features, neurodevelopmental delay, behavioral abnormalities, hearing impairment, seizures, and skeletal anomalies. The figure also outlines the diagnostic workflow of the presented case series, including clinical phenotyping, neuroimaging, and molecular confirmation.

## 5 Discussion

Consistent with previous reports, our patients demonstrated the characteristic combination of developmental delay, craniofacial dysmorphism, and behavioral abnormalities associated with KBG syndrome. Facial features, including a triangular face, synophrys, macrodontia, a long philtrum, and prominent ears, were identified across the cohort, aligning with previously reported phenotypic descriptions of the disorder (3, 16).

Macrodontia is a hallmark feature of KBG syndrome and is usually reported in the permanent dentition (16). Clinically evident macrodontia was observed in our patient's primary dentition. This may represent an unusual early dental manifestation; however, a panoramic dental x-ray was not available at the time of assessment.

From a neurological perspective, all patients exhibited global developmental delay involving both speech and motor milestones. Intellectual disability was observed in all cases, while autistic features were present with variable severity. Two patients fulfilled formal diagnostic criteria for autism spectrum disorder, whereas the third demonstrated autistic-like behaviors without a confirmed diagnosis. These findings are consistent with the established neurobehavioral manifestations reported in individuals with *ANKRD11* pathogenic variants (6, 17). The variability in neurodevelopmental severity observed within our cohort further supports previous evidence of significant inter-individual phenotypic heterogeneity in KBG syndrome (9, 17).

Skeletal manifestations were also prominent in our patients. Findings, including clinodactyly, scoliosis, delayed bone age, vertebral fusion anomalies, and butterfly vertebrae, are generally consistent with the recognized skeletal involvement described in KBG syndrome (4, 5, 18). Although vertebral anomalies have been previously reported, the complex axial skeletal abnormalities observed in our cohort may further expand awareness of the radiological variability associated with the disorder. These observations support recommendations for careful musculoskeletal and radiologic assessment in affected individuals. However, given the limited sample size, these findings should be interpreted cautiously and cannot establish definitive genotype–phenotype associations.

Renal involvement was identified in one patient with bilateral renal fusion anomaly. While renal abnormalities have been occasionally described in association with KBG syndrome, they remain less consistently recognized compared with the neurological and skeletal manifestations. Therefore, our findings may add to the existing evidence suggesting that renal anomalies could represent underrecognized features in some individuals with KBG syndrome. Further systematic studies are needed to determine their true frequency and clinical significance.

Auditory manifestations were observed in one patient who developed conductive hearing loss, consistent with previous reports describing hearing impairment as part of the multisystem involvement of KBG syndrome (9, 16). This finding further supports the importance of audiological surveillance during long-term follow-up, particularly in children with developmental and speech delay.

At the molecular level, all patients harbored heterozygous truncating variants in *ANKRD11*, including nonsense and frameshift variants. These findings are broadly consistent with haploinsufficiency being recognized as the principal pathogenic mechanism underlying KBG syndrome. Nevertheless, interpretation should remain cautious, as not all truncating variants are predicted to behave identically. Variants located within the 3′ region of *ANKRD11* may partially escape nonsense-mediated decay and could exert effects beyond simple dosage reduction (1). Functionally, *ANKRD11* interacts with nuclear receptor coactivators and histone deacetylases and plays an important role in transcriptional regulation and neural development (2).

The inclusion of patients across different age groups allowed limited observation of age-dependent phenotypic evolution. Consistent with previous studies, our findings suggest that KBG syndrome may remain underrecognized in early childhood, particularly when characteristic dysmorphic features are subtle or incomplete (10, 19). From a regional perspective, all patients originated from consanguineous families despite the autosomal dominant inheritance pattern of the disorder. Although this observation may reflect local population structure and referral patterns, no causal relationship can be inferred from this small cohort.

From a clinical standpoint, pediatricians and general practitioners should maintain awareness of KBG syndrome in children presenting with developmental delay, behavioral abnormalities, and subtle dysmorphic features, even in the absence of classic macrodontia. Early recognition and referral for genetic evaluation remain important to facilitate multidisciplinary management, surveillance for associated complications, rehabilitation support, and appropriate genetic counseling. Future larger multicenter studies with systematic neurological, skeletal, renal, and auditory evaluation are warranted to better define the full phenotypic spectrum and clinical variability of KBG syndrome across diverse populations.

### 5.1 Expected long-term clinical outcomes and prognosis

Long-term clinical outcomes in KBG syndrome remain variable and are primarily informed by previously published cohort studies and patient-reported surveys. Available data suggest that cognitive and behavioral manifestations may persist into adulthood, although severe intellectual disability is uncommon.

The clinical manifestations observed in our cohort illustrate the variable multisystem involvement and long-term support needs associated with KBG syndrome. Patient 1 demonstrated significant neurodevelopmental impairment with autism spectrum disorder features, conductive hearing loss, and complex partial seizure disorder, highlighting the importance of early neurological, behavioral, audiological, and rehabilitative interventions. His persistent developmental delay, impaired social interaction, aggressive behavior, and lack of independent daily living skills emphasize the substantial long-term functional impact that may persist into adolescence and the need for prolonged multidisciplinary care, structured behavioral support, and specialized educational services. Patient 2 exhibited developmental delay, hypotonia, autistic-like behaviors, and characteristic dysmorphic features without seizures, supporting previous observations that neurological involvement in KBG syndrome may range from mild learning difficulties to more significant intellectual and behavioral impairment. Early developmental therapies and close developmental follow-up remain important in such patients to optimize functional outcomes and improve adaptive functioning over time. Patient 3 presented with milder neurodevelopmental involvement but significant skeletal and renal manifestations, including scoliosis requiring spinal fixation and bilateral renal fusion anomaly, emphasizing the broad phenotypic spectrum of the disorder and the importance of systemic surveillance beyond neurological manifestations alone. Although her neurological investigations were unremarkable, the persistence of skeletal complications into adulthood highlights the potential for chronic orthopedic morbidity and the importance of long-term follow-up, multidisciplinary surveillance, and genetic counseling, particularly in the context of future family planning.

**Figure 4:**
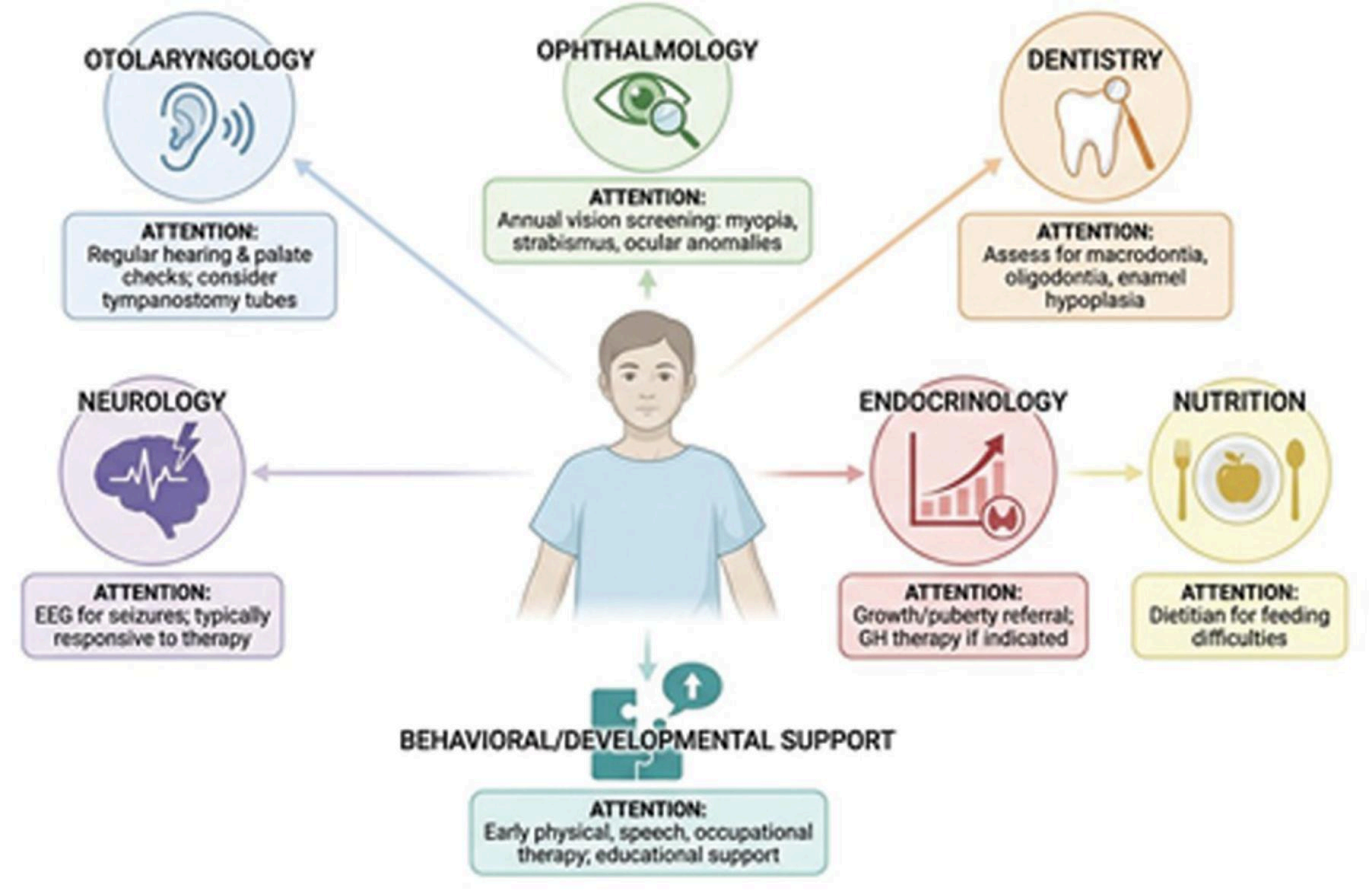
Image summarizing the comprehensive guide to multidisciplinary management, surveillance for associated complications, and lifelong care needs in KBG syndrome.

Overall, these findings add to existing evidence on phenotypic variability and highlight the importance of multidisciplinary management, surveillance for associated complications, and lifelong care needs for individuals with KBG syndrome to improve quality of life and functional outcomes (see Figure 4).

### 5.2 Further interventions and surveillance

- Otolaryngology: regular hearing assessments and palatal evaluation; tympanostomy tubes may be considered for recurrent otitis media.
- Ophthalmology: assessment for myopia, strabismus, and other ocular abnormalities (10).
- Dentistry: evaluation for macrodontia, oligodontia, and enamel hypoplasia (10).
- Neurology: EEG assessment when seizures are suspected; seizures generally respond well to treatment.
- Endocrinology: referral for short stature and pubertal concerns; growth hormone therapy may be considered in selected cases (13).
- Nutrition: referral to a dietitian and speech-language pathologist when feeding difficulties are present (11).
- Behavioral and developmental support: early physical, occupational, and speech therapy, as well as educational support for developmental and behavioral difficulties (12).

## 6 Limitations and recommendations

The small sample size limits the generalizability of the findings and precludes definitive genotype–phenotype correlation analysis. In addition, the retrospective single-center design may introduce selection and reporting bias, with some clinical data being incomplete or inconsistently documented. Limited longitudinal follow-up also restricts comprehensive assessment of disease progression, long-term outcomes, and age-dependent phenotypic evolution. Furthermore, functional validation studies were not performed; therefore, mechanistic interpretations regarding the identified variants remain limited. Some observed clinical findings, including renal and vertebral anomalies, may represent underrecognized associations; however, a coincidental coexistence of KBG syndrome with an unrelated congenital anomaly cannot be excluded. Therefore, this observation should be interpreted with caution and not considered a definitive expansion of the KBG phenotype without additional supporting cases or functional evidence.

Despite these limitations, our findings contribute to the existing evidence on the clinical heterogeneity of KBG syndrome and underscore the importance of comprehensive multisystem evaluation. Future multicenter studies with larger cohorts, standardized phenotyping approaches, longitudinal follow-up, and functional genomic investigations are needed to better clarify genotype–phenotype correlations, validate potentially underrecognized manifestations, and improve early clinical recognition and multidisciplinary management of affected individuals.

## 7 Conclusion

KBG syndrome is a rare neurodevelopmental disorder characterized by distinctive craniofacial features, macrodontia, skeletal anomalies, and developmental delay. Early clinical recognition supported by molecular confirmation of *ANKRD11* variants is essential for accurate diagnosis, particularly in individuals with subtle or atypical presentations.

From a clinical perspective, pediatricians and general practitioners should suspect KBG syndrome in children presenting with developmental delay and behavioral concerns in association with subtle dysmorphic features, even in the absence of classic findings such as macrodontia.

## Data Availability

The genetic variants reported in this study are available in publicly accessible databases. The relevant accession numbers are as follows: ClinVar, VCV000488985, available at https://www.ncbi.nlm.nih.gov/clinvar/variation/488985/; dbSNP, rs2544237769, available at https://www.ncbi.nlm.nih.gov/snp/rs2544237769/; and dbSNP, rs749201074, available at https://www.ncbi.nlm.nih.gov/snp/rs749201074/.
